# On the Inside

**DOI:** 10.1093/plphys/kiab218

**Published:** 2021-07-04

**Authors:** Peter V Minorsky

**Affiliations:** School of Health and Natural Sciences, Mercy College, Dobbs Ferry, NY, USA

## N-hydroxypipecolic acid and systemic acquired resistance

The immune responses of previously unchallenged plants are generally not effective enough to entirely prevent infection by well-adapted pathogens. However, a localized leaf inoculation can induce systemic acquired resistance (SAR) in the foliage of the whole plant. Plants with activated SAR show broad-spectrum immunity against further microbial infestation and are primed for a timely and boosted induction of defenses in response to pathogens. The establishment of SAR requires an interplay of two immune-regulatory metabolites, salicylic acid (SA) and N-hydroxypipecolic acid (NHP), which both accumulate to substantial levels in inoculated and in distant, noninoculated leaves of pathogen-attacked plants. Previous studies have revealed that the inoculation of Arabidopsis (*Arabidopsis thaliana*) with pathogens systemically activates an SAR-related transcriptional reprogramming as well as a primed immune status that is strictly dependent on FLAVIN-DEPENDENT MONOOXYGENASE 1 (FMO1), an enzyme that mediates the biosynthesis of NHP. Yildiz et al. (pp. 1679–1705) now show that exogenously applied NHP is sufficient to induce upregulation of more than 1,500 SAR-related genes in Arabidopsis and primes plants for an enhanced pathogen-triggered activation of defense metabolism. Primed metabolic responses included the biosynthesis of SA, pipecolic acid (a precursor of NHP), and branched-chain amino acids, as well as the accumulation of the phytoalexin camalexin. NHP also conditioned Arabidopsis for effective SA- and pathogen-induced expression of defense-related genes. All the effects of exogenous NHP require the function of the transcriptional coregulator NONEXPRESSOR OF PR GENES1 (NPR1). These results suggest that NPR1 transduces NHP-activated immune signaling modes that involve predominantly SA-dependent and, to a lesser extent, SA-independent features. The authors propose that NHP functions as a mobile immune regulator capable of moving between leaves to systemically activate immune responses.

## A new tool in the fight against parasitic plants

Root parasitic weeds, including witchweeds (*Striga* spp.) and broomrapes (*Orobanche* and *Phelipanche* spp.), are a major biological threat to global food security, affecting cereal crops in sub-Saharan Africa, and noncereal crops in Central Asia and the Mediterranean area. A single parasitic plant can produce tens of thousands of tiny and highly viable seeds that add to a seemingly inexhaustible seed bank in the soil. The life cycle of these parasitic weeds begins with seed germination that requires—in contrast to nonparasitic plants—chemical stimulants, mainly strigolactones (SLs), released by host plants. (These SLs are released by the host to establish symbiosis with arbuscular mycorrhizal fungi under nutrient-deprived conditions.) From an agricultural perspective, this unusual requirement for SLs is possibly the Achille’s heel of these parasitic weeds: it may be possible to control them by applying specific germination inhibitors or synthetic stimulants that induce lethal germination in the host’s absence. Current chemical screening technologies, unfortunately, are labor intensive. To address this problem, Braguy et al. (pp. 1632–1644) have developed an automatic seed census tool to count and discriminate germinated from nongerminated seeds. This new method shows an accuracy of 94% in counting seeds of *Striga hermonthica* and reduced the required time from 5 min to 5 s per image. The open-source software the authors have developed, SeedQuant, will enable high-throughput screening for germination stimulants/inhibitors, and can also be used to study the germination of nonparasitic plant species.

## Screening stomatal traits in sorghum

Stomata, the microscopic pores that occur on the surfaces of leaves, have a huge effect on crop productivity by influencing photosynthesis and water use. Stomatal traits, therefore, are potential targets for crop improvement. Sorghum (*Sorghum bicolor*) is generally grown in arid and semi-arid regions, and hence its productivity depends on timing and amount of rainfall. Thus, exploring the phenotypic diversity and genetic basis of stomatal traits would provide useful information to improve productivity and stress tolerance in sorghum. Stomatal size/complex area (SCA), the product of guard cell length and width, and stomatal density (SD) are two key parameters regulating gas exchange and abiotic stress response in many plants, but sorghum remains to be studied in this regard. Moreover, the diversity in stomatal traits is largely unexplored or utilized in breeding programs in general, due to cumbersome phenotyping protocols that require substantial investment of resources. Bheemanahalli et al. (pp. 1562–1579) now report success in characterizing the phenotypic diversity of 311 sorghum accessions for stomatal traits grown under two different field environments. The nearly 12,000 images collected from abaxial and adaxial leaf surfaces revealed substantial variation in stomatal traits. In sorghum, SD was 32%–39% greater on the abaxial vs. the adaxial surface, while SCA on the abaxial surface was 2%–5% lower than on the adaxial surface. By using a Genome-Wide Association Study, a powerful tool for unraveling the genetic basis of complex traits compared to biparental mapping, the authors succeeded in identifying 71 genetic loci underlying SCA and SD in sorghum. These findings provide the foundation for further studies on the genetic and molecular mechanisms controlling stomatal regulation and patterning in sorghum.

## Systemic reactive oxygen species signaling

The local perception of heat (HS), high light (HL), or mechanical injury by a single leaf triggers systemic effects, such as systemic acquired acclimation or systemic wound responses, which induce a heightened state of stress readiness in the whole plant. Among the main candidates for the systemic signals mediating these responses are electric, calcium, and reactive oxygen species (ROS). These signals propagate from the stressed or injured leaf to the rest of the plant through the plant vascular bundles. It is thought that during this process the different cells along the path of signaling chain are activated one-by-one starting at the initial (local) tissue and ending at the systemic tissue and that this activation process propagates and maintains the different systemic signals. It was found in Arabidopsis, for example, that the ROS produced by each cell during this process is generated by the RESPIRATORY BURST OXIDASE HOMOLOG D (RBOHD) protein and that this process is controlled by calcium-dependent activation of RBOHD. The ROS being used as a systemic signal, most likely H_2_O_2_, is therefore actively generated by each cell along the path of the signal, as opposed to being made in the local tissue and somehow transported over long distances. However, whether these signals can propagate through other cell types, and whether they are interlinked, remain open questions are the topic of a contribution by Zandalinas and Mittler (pp. 1721–1733). Their findings reveal that in contrast to RBOHD-dependent systemic responses to HL stress, which were exclusively mediated through the vascular bundles of Arabidopsis, RBOHD-dependent systemic signaling during HS, or wounding, are mediated through the vascular bundles and/or mesophyll cells. They further show that propagation of the ROS wave through mesophyll cells could contribute to the stronger systemic ROS signal observed in plants subjected to HL and HS applied simultaneously to two different leaves. These findings demonstrate that ROS production in mesophyll cells is required for the propagation of rapid systemic ROS signals during responses to HS or wounding.

## A heat shock protein involved in embryogenesis

The formation of the main body axis is a critical first step in the embryological development of plants. During its initial asymmetric division, the zygote partitions into a small apical daughter cell and a large vacuolated basal daughter cell. The apical cell gives rise eventually to the proembryo, while the basal cell gives rise to the suspensor that connects the embryo to maternal tissue. The YODA kinase (YDA) pathway is one of the major signaling cascades controlling cell division plane orientation and cell division symmetry. It acts by targeting and phosphorylating transcription factors involved in specific developmental processes. *YDA* encodes an MPK kinase involved in zygote elongation and the shootward extra-embryonic cell fate. Loss-of-function *yda* zygotes do not elongate and after the first asymmetric division, a short basal cell with a repressed suspensor cell fate is produced. Conversely, constitutive activation of *YDA* leads to opposite phenotypes manifested by very long suspensors and in extreme cases the apical cell fate is fully suppressed. By means of genetic analysis, Samakovli et al. (pp. 1526–1544) now report that HEAT SHOCK PROTEIN 90 (HSP90) proteins are potent regulators of YDA during embryo development and patterning. The genetic interactions of HSP90 with YDA affected the downstream signaling pathway to control the development of both basal and apical cell lineage of embryo. Their results demonstrate that the spatiotemporal expression of WUSCHEL-RELATED HOMEOBOX8 (WOX8) and WOX2, two major players in axis formation, is changed when the function of HSP90s or YDA is impaired, suggesting their essential role in the cell fate determination and possibly a link to auxin signaling during early embryo development. Hence, HSP90s together with the YDA signaling cascade affect transcriptional networks shaping early embryo development. Thus, HSP90s are new players involved in the main body axis formation during early embryogenesis in Arabidopsis.

## Cavitation fatigue in conifers

Coping with drought is fundamentally important for tree growth and survival. Water shortage in trees impairs the long-distance water transport when critical negative xylem pressure induces cavitation and, consequently, xylem embolism and disruption of the continuity of water columns. Many cases of drought-related forest mortality have already been reported and such events are likely to become more frequent, as increases in duration, intensity, and frequency of dry spells, coupled with rising temperatures, are predicted to occur in the future. After drought-induced embolism and repair, tree xylem may be weakened against future drought events (cavitation fatigue). Feng et al. (pp. 1580–1590) have quantified vulnerability curves after embolism/repair cycles in eight European conifer species. All species demonstrated complete embolism repair and a lack of any cavitation fatigue after 50% loss of conductivity (LC). After 100% LC, European larch (*Larix decidua*), stone pine (*Pinus cembra*), Norway spruce (*Picea abies*), and silver fir (*Abies alba*) remained unaffected, while mountain pine (*P. mugo*), yew (*Taxus baccata*), and common juniper (*Juniperus communis*) exhibited 0.4–0.9 MPa higher vulnerability to embolism. These data demonstrate that cavitation fatigue in conifers is species-specific and depends on the intensity of the preceding LC. The lack of fatigue effects after moderate LC, and relevant effects in only three species after high LC, indicate that conifers are relatively resistant against cavitation fatigue. This is remarkable considering the complex and delicate conifer pit architecture and may be important considering climate change projections.

